# Does the transverse acetabular ligament‐guided cup anteversion fulfil the functional requirements in total hip arthroplasty?

**DOI:** 10.1002/jeo2.70454

**Published:** 2025-10-13

**Authors:** Zhuyi Ma, Xiang‐Dong Wu, Yixin Zhou, Hao Tang, Hongyi Shao, Yunfeng Zhang

**Affiliations:** ^1^ Department of Orthopaedic Surgery, Beijing Jishuitan Hospital, Capital Medical University, Fourth Clinical College of Peking University National Center for Orthopaedics Beijing China; ^2^ Beijing Research Institute of Traumatology and Orthopaedics, Beijing Jishuitan Hospital, Capital Medical University, Fourth Clinical College of Peking University National Center for Orthopaedics Beijing China

**Keywords:** functional requirements, kinematic alignment, patient‐specific safe zone, total hip arthroplasty, transverse acetabular ligament

## Abstract

**Purpose:**

The transverse acetabular ligament (TAL) plays a pivotal role in determining cup anteversion during total hip arthroplasty (THA). Meanwhile, the kinematic alignment (KA) technique focuses on restoring the native hip anatomy and adjusting cup orientation based on the TAL. This study aims to investigate whether utilizing the TAL or TAL‐based KA as a guide for aligning cup anteversion satisfies the functional requirements of THA.

**Methods:**

This retrospective study included 92 patients (107 hips; 40 men and 52 women). TAL anteversion was assessed using computed tomography scans. Radiographic inclination angles were standardized at 40°. The analysis involved comparing the ratios of TAL and TAL‐based KA to determine their alignment with the criteria of the Lewinnek safe zone (LSZ), combined sagittal index (CSI) ante‐inclination (AI) criteria, and patient‐specific safe zone (PSSZ).

**Results:**

The mean TAL anatomical anteversion (AA) angle was 18.90 ± 5.56°. No significant difference was observed between the left and right sides (*p* = 0.185). Men had an average TAL AA angle of 17.26 ± 5.09°, whereas women had 20.29 ± 5.61°, indicating a significant difference in TAL anteversion between sexes (*p* = 0.005). For TAL, 104 cups (97.2%) were aligned according to the LSZ criteria, while 54 hips (50.47%) met the CSI AI criteria. The PSSZ criteria were met by 20 hips (18.69%). The distribution of TAL‐based KA was consistent with these results.

**Conclusions:**

The TAL or TAL‐based KA for aligning cup anteversion does not consistently satisfy the functional requirements of THA.

**Level of Evidence:**

Level III.

**Abbreviations**
3D: three‐dimensional
AA: anatomical anteversion
AI: ante‐inclination
APP: anterior pelvic plane
ASIS: anterior superior iliac spines
CSI: combined sagittal index
CT: computed tomography
IFSZ: Impingement‐free safe zone
KA: kinematic alignment
LSZ: Lewinnek safe zone
OA: operative anteversion
PSSZ: patient‐specific safe zone
RA: radiographic anteversion
RI: radiographic inclination
ROM: range of motion
SHR: spinal‐hip relationship
TAL: transverse acetabular ligament
THA: total hip arthroplasty

## INTRODUCTION

Suboptimal cup anteversion in total hip arthroplasty (THA) can lead to impingement, dislocation, and wear [22, 23]. Archbold et al. [2] introduced a technique utilizing the transverse acetabular ligament (TAL) to determine cup anteversion during THA, aiming to reduce the dislocation rate. Although the TAL is widely used as a reference for anteversion, its effectiveness in fulfilling THA post‐surgical functional requirements remains uncertain [4, 16]. Rivière et al. [19] proposed the kinematic alignment (KA) technique for THA, focusing on restoring the native hip anatomy, adjusting cup orientation based on the TAL, and accounting for abnormal spinal‐hip relationships (SHRs) [20, 21]. SHRs were categorized using the Simplified Bordeaux classification, consisting of six types (A, 1, B, C, D, and F). The Type A lumbo‐pelvic complex is flexible, negating the need to adjust the cup anteversion angle. Types 1 and B pertain to individuals who sit without sufficiently retroverting their pelvis; thus, cup anteversion must be increased by 3.5° (relative to TAL) for every 10° lack of pelvic retroversion when sitting. Types C and D are associated with an ageing, stiff, degenerated spine, fixing the pelvis in a chronically retroverted position when standing; thus, cup anteversion must be reduced by 3.5° (relative to TAL) for every 10° of excessive standing pelvic retroversion. For Type F (fused spine), following the recommendations for either Type B or C depends on the fusion position and remaining flexibility. Further research is needed to assess the effectiveness of the KA technique.

Recently, the role of spinal‐pelvic motion in hip stability has gained increasing attention. Lewinnek et al. [11] initially proposed the concept of a safe zone for acetabular cups in 1978, known as the Lewinnek safe zone (LSZ). However, increasing evidence indicates that the LSZ is not a reliable predictor of dislocation after THA, with many dislocation cases occurring in patients whose cup prostheses are positioned within the LSZ [3]. As a substitute, the combined sagittal index (CSI) was introduced, representing the sum of the ante‐inclination (AI) and pelvic femoral angle, which is measured in both standing and sitting positions [9, 10]. The AI reflects the combined effects of anteversion and inclination of the acetabular component. The normal AI range is defined as follows: the upper limit of standing AI is 45°, and the lower limit of sitting AI is 41° [26]. CSI represents substantial progress in optimal functional cup alignment in THA.

With progress in three‐dimensional (3D) planning, the PSSZ has evolved into an impingement‐free safe zone (IFSZ) from standing to sitting, as suggested by Tang et al. [24, 25]. This 3D approach optimizes cup and stem orientations to provide precise target angles of the cup for a specific patient, aiming to minimize the risks of dislocation and wear. Critical factors influencing this include standing and sitting pelvic tilt (PT), head diameter, range of motion (ROM) criteria, stem version, anterior pelvic plane (APP), the centre of the superior sacral endplate, and normal bilateral hip rotation centres [24].

Currently, the assessment of TAL effectiveness relies on the LSZ criteria. However, the LSZ may not entirely encompass a functionally safe zone. It remains uncertain how effectively the TAL or TAL‐based KA aligns with the dynamic functional safe zones of the CSI and PSSZ. Therefore, we questioned, ‘What was the incidence of anteversion determined by the TAL falling into the new safe zones of CSI and PSSZ? Was it possible to adjust the TAL using the KA technique to meet functional requirements better?’ Our study aimed to investigate whether the application of TAL or TAL‐based KA for aligning cup anteversion satisfied the functional requirements of THA.

## METHODS

### Patient selection

Implementation of this study was approved by our Institutional Review Board (No. K2023205‐00), and all methods were performed in accordance with the Declaration of Helsinki and its later amendments. The need for informed consent was waived due to the retrospective nature of the study. A total of 92 patients who underwent robotic‐assisted THA between August 2019 and December 2020 were eligible for inclusion, with their main characteristics presented in Table 1. Exclusion criteria included patients with posttraumatic malunion, prior periacetabular or femoral osteotomy, acetabular protrusion, severe developmental hip disease, or severely stiff or degenerated hips. Additionally, patients with incomplete imaging data or substantial osteophyte hyperplasia, complicating the TAL identification, were excluded. All arthroplasties were performed by an experienced orthopaedic surgeon using a modified Gibson approach, a tritanium acetabular component (Stryker), and an Accolade II stem (Stryker). All patients underwent preoperative computed tomography (CT) and EOS (Biospace) imaging in standing and sitting positions.

**Table 1:** Characteristics of the participants.

Baseline characteristics	*N*
Number of patients (hips viewed)	92 (107)
Mean age (range)	49.9 (21–78)
Sex (men/women)	40/52
Sex of hips measured (men/women)	49/58
Side of hips measured (left/right)	51/56

### TAL measurements

**Figure 1:**
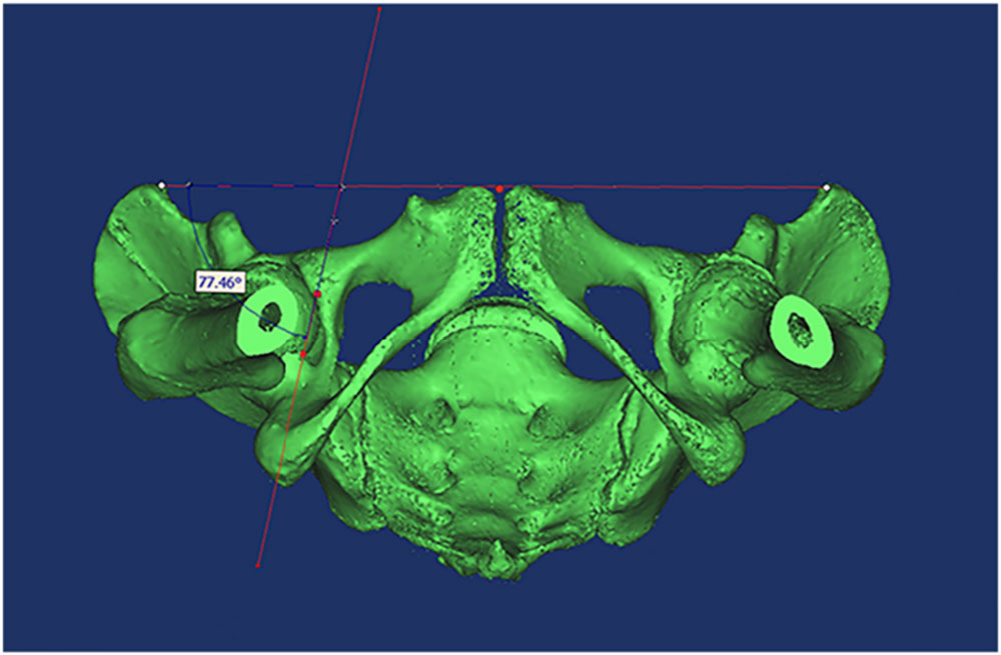
Measurements of the AA of the TAL with the APP as the reference plane. The 3D CT was adjusted to ensure the APP was perpendicular to the observer in a straight line. The midline of the sacrum was located at the centre of the pubic tubercles, and the bilateral obturator foramina were equal in size. The residual angle between the TAL and the bilateral ASIS lines was defined as the AA of the TAL. 3D, three‐dimensional; AA, anatomical anteversion; APP, anterior pelvic plane; CT, computed tomography; TAL, transverse acetabular ligament.

Preoperatively, a spiral CT scanner (80‐slice CT scanner; Aquilion Prime, Toshiba) was used to scan the entire pelvis. Mimics software 17 (Materialise) was used for 3D analysis. The APP established through the bilateral anterior superior iliac spines (ASIS) and pubic tubercles was adopted as the reference coronal plane to measure anatomical anteversion (AA) of the TAL. The TAL, defined as a line on 3D CT scans connecting the posteroinferior and anteroinferior edges of the acetabular rim across the acetabular notch, was analyzed [1]. Adjustments were then made to view the 3D CT image from a bottom perspective, aligning the APP perpendicularly to the observer. This ensured the sacral midline was in line with the midpoint of the pubic tubercles and achieved bilateral symmetry of the obturator region. The TAL anteversion angle was defined as the angle between the TAL and the perpendicular line of the APP projected onto the vertical plane of the APP (Figure 1). For each TAL angle, two measurements were taken, and the average value was recorded.

### Adjusting the TAL orientation according to the KA technique

According to the analysis of lateral lumbopelvic images in both standing and sitting positions, patients were categorized into SHR A (82 hips, 76.64%), 1 (6 hips, 5.61%), B (5 hips, 4.67%), C (7 hips, 6.54%), D (5 hips, 4.67%), or fused spine (2 hips, 1.87%).

To accommodate individual SHR variations, a total of 11 TAL anteversion angles were adjusted using previously described methods. All radiographic inclinations (RIs) were standardized at 40°, based on the KA technique. Following the formula established by Murray et al. [15], AA was transformed to obtain radiographic anteversion (RA) of the TAL.

Tan(AA)=Tan(RA)/Sin(RI).

### Using PSSZ to verify if the TAL anteversion is in the safe zone

**Figure 2:**
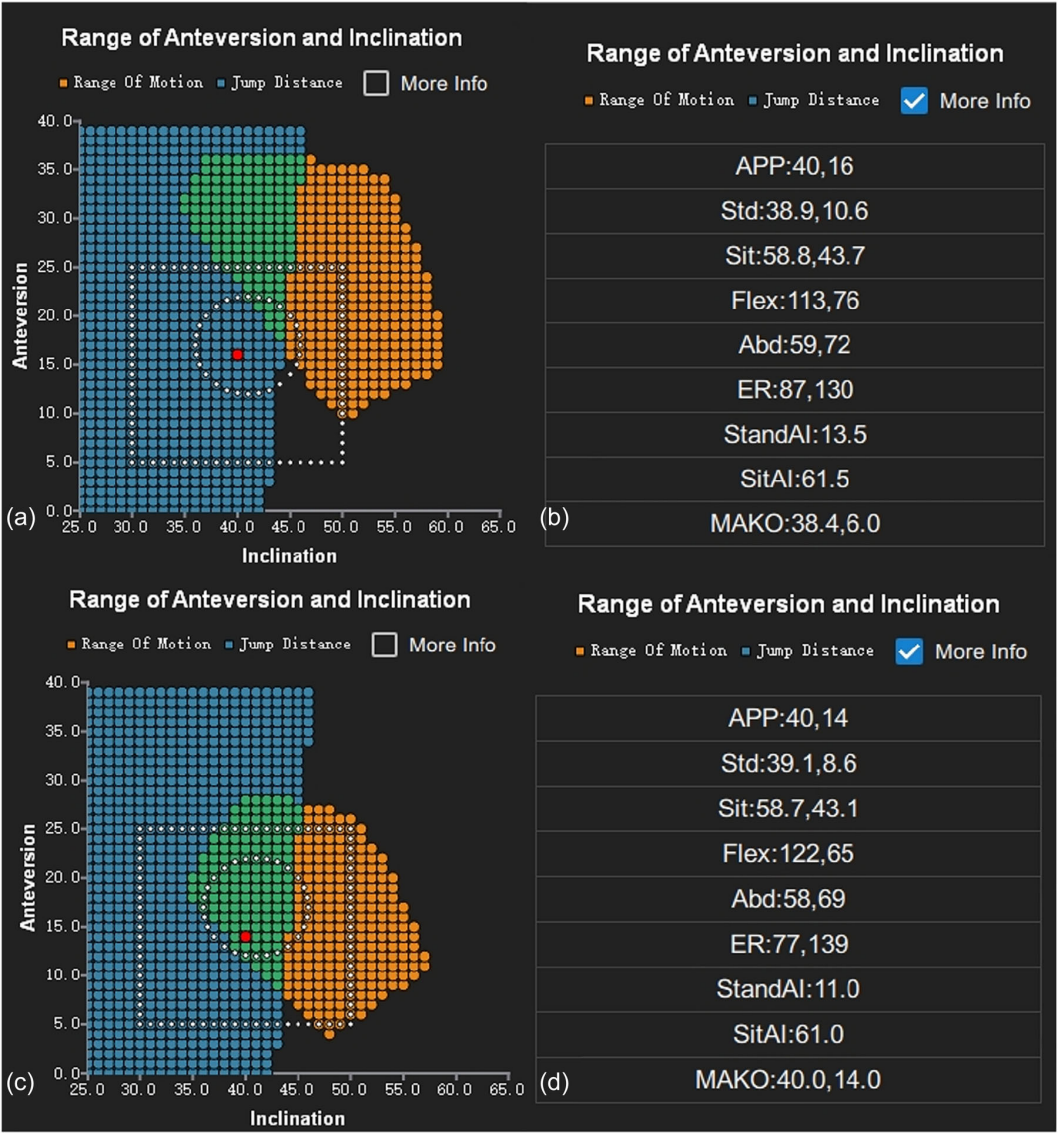
The algorithm of PSSZ could provide the LSZ, AI and PSSZ criteria. (a) The green area in the figure represents the PSSZ criteria, while the square box indicates the LSZ criteria. The red point signifies the target angle, which fell within the LSZ criteria but not the PSSZ criteria. (b and d) The algorithm of PSSZ could provide predicted post‐operative AI for each point. (c) The red point is judged to be within the LSZ and PSSZ criteria. AI, ante‐inclination; LSZ, Lewinnek safe zone; PSSZ, patient‐specific safe zone.

For each patient, we calculated the PSSZ algorithm using the information after THA from the application, considering the actual diameter of the femoral stem head. We established a unified expected post‐operative ROM for each patient. If the Mako robot provided the post‐operative stem version, it was used; otherwise, it was measured based on post‐operative CT or EOS imaging [12]. Calculations were also performed based on a hypothetical stem anteversion angle of 15°. Prior to surgery, the modelling team segmented the CT scans, thereby identifying the APP, the superior centre of S1, and the bilateral hip rotation centres. Due to the absence of post‐operative bi‐dimensional images, preoperative images in functional standing and sitting positions were used to measure standing and sitting PT. The software calculated the angle using an APP reference. The PSSZ criteria, depicted in the figure, were defined by the intersecting orientations of the standing and sitting IFSZs, excluding points where the standing functional acetabular cup RI exceeded 45°. The square box in the figure indicates the LSZ (Figure 2a,c). The PSSZ algorithm predicted the post‐operative AI for each point (Figure 2b,d). By setting the RI and calculating RA, we identified the point corresponding to the TAL and TAL‐based KA. Therefore, by comparing the angle proportions provided by the TAL and KA technique with the LSZ, AI, and PSSZ criteria, we assessed the alignment of the TAL and KA technique with the functional requirements.

### Data analyses

Descriptive statistics included means and standard deviations (mean ± SD). Normality was assessed using the Shapiro–Wilk test. The TAL AA was analyzed for differences in sex and side using an independent‐samples *t*‐test (two‐tailed). A *p* value of <0.05 was considered statistically significant. IBM SPSS Statistics version 26.0 (IBM) was used to conduct the statistical analysis.

## RESULTS

**Figure 3:**
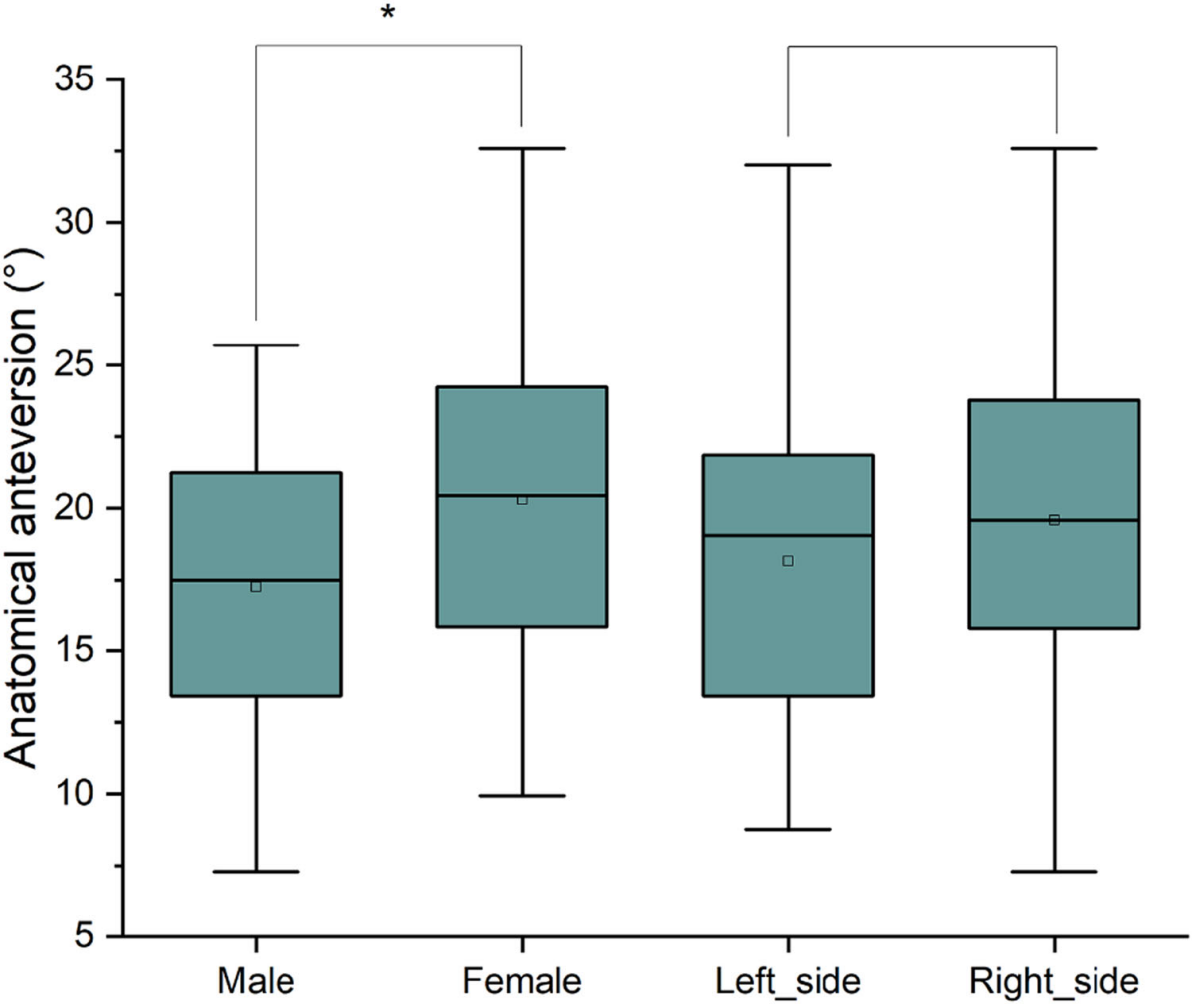
The AA of the TAL was compared for sex and side differences. The AA of the TAL was significantly higher in women than men (*p* = 0.005). There was no significant difference between the left and right sides (*p* = 0.185). AA, anatomical anteversion; TAL, transverse acetabular ligament.

The TAL and TAL‐based KA anteversion showed a normal distribution. The mean TAL AA angle was 18.90 ± 5.56°. The mean AA angles for the left and right sides were 18.15 ± 5.29° and 19.58 ± 5.77°, respectively (Table 2). No significant differences were observed between the left and right AA angles (*p* = 0.185) (Figure 3). The mean TAL RA angle was 12.48 ± 3.83°. The mean RA angles for the left and right sides were 11.96 ± 3.62° and 12.96 ± 3.99°, respectively. After adjusting the cup orientation according to the KA technique, the mean KA AA angle was 19.16 ± 5.84°. The mean left and right side KA AA angles were 18.22 ± 5.50° and 20.01 ± 6.06°, respectively. The mean KA RA angle was 12.67 ± 4.04°. The mean KA RA angles for the left and right sides were 12.01 ± 3.79° and 13.26 ± 4.21°, respectively (Table 2).

**Table 2:** Summary of anteversion angle (°).

	TAL AA	TAL left AA	TAL right AA	TAL men AA	TAL women AA	TAL RA	TAL left RA	TAL right RA	KA AA	KA left AA	KA right AA	KA RA	KA left RA	KA right RA
Mean	18.90	18.15	19.58	17.26	20.29	12.48	11.96	12.96	19.16	18.22	20.01	12.67	12.01	13.26
SD	5.56	5.29	5.77	5.09	5.61	3.83	3.62	3.99	5.84	5.50	6.06	4.04	3.79	4.21

*Note*: Mean ± SD.

Abbreviations: AA, anatomical anteversion; KA, kinematic alignment; RA, radiographic anteversion; SD, standard deviation; TAL, transverse acetabular ligament.

There was a significant difference in the TAL anteversion angles between men and women (*p* = 0.005) (Figure 3). The mean TAL AA angles for men and women were 17.26 ± 5.09° and 20.29 ± 5.61°, respectively (Table 2).

Additionally, the mean TAL standing and sitting AI angles were 8.98 ± 6.99° and 41.54 ± 13.44°, respectively. After adjusting the TAL angle using the KA technique, the mean KA standing and sitting AI angles were 9.19 ± 7.09° and 41.69 ± 13.08°, respectively (Table 3).

**Table 3:** Summary of AI angle (°).

	TAL standing AI	TAL sitting AI	KA standing AI	KA sitting AI
Mean	8.98	41.54	9.19	41.69
SD	6.99	13.44	7.09	13.08

*Note*: Mean ± SD.

Abbreviations: AI, anatomical inclination; KA, kinematic alignment; SD, standard deviation; TAL, transverse acetabular ligament.

The proportion of cup placement adjusted by TAL or TAL‐based KA matching the LSZ, functional safe zone based on CSI and PSSZ gradually decreased. Concerning the LSZ criteria, 104 cups (97.2%) based on TAL were deemed within the safe zone (left side, 100%; right side, 94.64%). The TAL‐based KA exhibited the same proportion. Regarding the AI criteria, 54 cups (50.47%) met the safety criteria (left side, 50.98%; right side, 50%), reflecting a similar proportion of TAL‐based KA. For the PSSZ criteria, based on the post‐operative stem anteversion, 20 cups (18.69%) complied with the safe zone (left side, 21.57%; right side, 16.07%). The TAL‐based KA had the same proportion. Considering a set stem anteversion of 15°, 26 cups (24.3%) were found within the safe zone (left side, 29.41%; right side, 19.64%) for TAL. Meanwhile, 27 cups (25.23%) were within the safe zone (left side, 31.37%; right side, 19.64%) for TAL‐based KA.

## DISCUSSION

The results revealed that while the proportion of cup placements adjusted by TAL or TAL‐based KA aligned with the LSZ, the functional safe zones based on CSI and PSSZ remained consistent and gradually decreased. This indicated that TAL and KA techniques could not fully meet the functional requirements. Additionally, it was observed that women had significantly higher anteversion angles than men when measured by TAL, with no significant difference between the left and right sides. This finding aligned with those from previous studies [6, 8, 13].

Many surgeons have adopted the TAL as a key anatomical landmark for cup anteversion in non‐navigated THA. Fukui et al. [6] conducted a simulated THA using an image‐free navigation system, during which a trial cup was inserted and snugly fitted onto the TAL. The measured RA averaged 18.2 ± 7.2° (range, 2.0–33.2°), and 26 cups (80.6%) fell within the LSZ. They concluded that the TAL was an effective intraoperative landmark for aligning the acetabular components. However, the reliability and applicability of these findings were limited by the small sample size. Meermans et al. [13] conducted a prospective, randomized, controlled trial to compare the freehand technique with the TAL as a reference. They found that 22.5% of the prostheses in the freehand group were located outside the LSZ, whereas none were in the TAL group, indicating that the TAL could significantly contribute to achieving the desired anteversion. Nonetheless, given the growing scepticism regarding the accuracy of the LSZ due to dynamic spinal‐pelvic interactions, re‐evaluating the strategy of utilizing the TAL to achieve optimal cup orientation is necessary.

While TAL anteversion generally aligns with the LSZ, individual variations exist and are influenced by factors such as sex, disease, and other related aspects. Additionally, some TALs may be obscured by soft tissue or osteophytes, making the TALs difficult to visualize and prone to destruction when removing the osteophytes [17]. Researchers have also examined the reliability of the TAL as a reference in patients with abnormal acetabular morphology, such as developmental dysplasia of the hip. The results and conclusions regarding the suitability of the TAL in such cases are controversial. Some researchers argue that TAL lacks reliability as a guide for hips with dysplasia [1]. However, other authors suggest that the TAL is a practical anatomical landmark for determining cup anteversion in both dysplastic and non‐dysplastic hip cases [5, 14]. The exclusion of dysplastic and other structurally altered hips was intended to minimize measurement variability and anatomical confounding factors, ensuring reliable assessment of TAL‐based alignment in anatomically preserved hips. Future studies should extend analyses to high‐risk populations by incorporating advanced 3D reconstruction and dynamic SHR assessment techniques, such as EOS imaging, to comprehensively evaluate TAL‐guided cup orientation in these challenging cases. Ning et al. [17] proposed that the TAL is a preferable landmark for patients with hip ankylosis, because bone fusion in the hip is primarily located in the upper and outer weight‐bearing areas.

With the APP as a reference, the TAL anteversion angle remained constant. However, the PT fluctuates with the patient's movements during daily activities. Additionally, SHR affects how PT varies when transitioning from different positions. The KA technique, which integrates anatomical hip reconstruction with kinematic cup alignment, facilitates near‐physiological periprosthetic soft tissue equilibrium and minimizes the risk of suboptimal dynamic component interaction during daily activities [19]. Rivière et al. [20] conducted a matched case‐control study using prospectively collected clinical data and reported favourable short‐term safety outcomes of KA‐guided THA, with no complications such as dislocation observed during a 1‐year follow‐up period. This evidence supports the potential clinical value of adjusting TAL orientation via the KA technique to achieve optimized acetabular anteversion from a functional perspective, which further supports the rationale behind incorporating the KA technique in the current analysis.

Dorr et al. [3, 26] have shown that spinal‐pelvic motion plays a pivotal role in determining the functional orientation of components, rendering the LSZ ineffective for predicting dislocations. The PSSZ algorithm, introduced by Tang et al. [24, 25], presents an effective method for calculating the IFSZ by considering both standing and sitting PT, significantly reducing the dislocation rate post‐THA. Tang et al. [25] employed CSI to highlight the limitations of the traditional LSZ in preventing dislocations post‐THA, noting that about one‐third of the LSZ failed to meet the CSI criterion. Consequently, we adopted the CSI AI and PSSZ as the reference safe zones. Our findings revealed that only half and one‐quarter of the cups aligned with the LSZ met the CSI and PSSZ criteria, respectively, suggesting that the guidance of the TAL or TAL‐based KA for aligning cup anteversion might not meet the functional demands of THA. Nonetheless, it is crucial to recognize that the small percentage of cup placements satisfying the PSSZ criteria may not be evident in clinical practice. This is attributed to patients typically avoiding extreme movements, and impingement does not always result in noticeable symptoms, such as dislocation, underscoring the challenge of attaining optimal functional cup orientation during THA [7, 18].

Our study found that if the set stem anteversion was 15°, the proportion within the PSSZ was higher. We suggested that stems with an adjustable version were more consistent with the physiological requirements. However, the increase in this proportion was not statistically significant and thus requires further verification.

Our study had some limitations. The classification of KA requires a physical examination. However, our assessment was based on medical records and full‐length EOS scans to determine the presence of severe fixed‐flexion hip deformity. In studies on cup orientation, it is essential to consider the three definitions proposed by Murray [15]. In actual surgical procedures, the KA technique should be applied for operative anteversion (OA) of the TAL. In our study, we made adjustments to the AA due to measurement constraints. Considering that the KA technique aims to restore the native hip anatomy and the adjustment angle is relatively small, with a standardized RI of 40°, the average of the 11 adjusted TAL AA anteversions is 24.32 ± 6.54°, while the mean converted RA is 16.31 ± 4.61°. When converted to OA, the average angle is 21.11 ± 6.01°, with the converted RA measuring 16.54 ± 4.83° (*p* = 0.105). We contend that modifying the AA or OA did not significantly impact the outcomes. Moreover, the small number (*n* = 11) of TAL anteversions adjusted using the KA technique limited the statistical robustness of conclusions derived from this subgroup. Given our study's descriptive rather than hypothesis‐driven approach, a formal power analysis was not applicable in this context. Future studies should aim to expand the cohort size and incorporate a formal power analysis during the study‐design phase, particularly when planning hypothesis‐driven comparisons across different SHR subgroups. Another limitation of this study was its reliance solely on retrospective image analysis without direct correlation to functional or clinical outcomes such as dislocation, revision rates, or patient‐reported outcomes. Thus, the clinical relevance of the observed mismatches between our radiological findings and the CSI/PSSZ criteria remained theoretical. Future prospective studies are warranted to validate these findings by correlating radiological alignment directly with patient‐centred functional outcomes.

## CONCLUSION

This study found that the TAL or TAL‐based KA failed to accurately align with the functional orientation of the acetabular cup. The proportion of cup placements guided by TAL or TAL‐based KA that aligned with the LSZ and the functional safe zone (based on CSI and PSSZ) decreased.

## AUTHOR CONTRIBUTIONS

All authors were involved in the drafting of this article or the critical revising for the important intellectual content, and all authors approved the final version to be published. All authors had full access to all the data in the study and are responsible for the integrity of the data and the accuracy of data analysis. *Conceptualization*: Zhuyi Ma, Hao Tang and Hongyi Shao. *Data curation and Formal analysis*: Xiang‐Dong Wu and Yunfeng Zhang. *Writing—original draft*: Zhuyi Ma. *Writing—review and editing*: Zhuyi Ma, Xiang‐Dong Wu and Yixin Zhou.

## CONFLICT OF INTEREST STATEMENT

The authors declare no conflicts of interest.

## ETHICS STATEMENT

Implementation of this study was approved by Beijing Jishuitan Hospital's Institutional Review Board (No. K2023205‐00), and all methods were performed in accordance with the Declaration of Helsinki and its later amendments. The need for informed consent was waived due to the retrospective nature of the study.

## Data Availability

The datasets generated and analyzed during the current study are available from the corresponding author upon reasonable request.
